# Ellipsometry and XPS comparative studies of thermal and plasma enhanced atomic layer deposited Al_2_O_3_-films

**DOI:** 10.3762/bjnano.4.83

**Published:** 2013-11-08

**Authors:** Jörg Haeberle, Karsten Henkel, Hassan Gargouri, Franziska Naumann, Bernd Gruska, Michael Arens, Massimo Tallarida, Dieter Schmeißer

**Affiliations:** 1 Brandenburg Technical University, Applied Physics and Sensors, K.-Wachsmann-Allee 17, 03046 Cottbus, Germany; 2 Sentech Instruments GmbH, Schwarzschildstraße 2, 12489 Berlin, Germany

**Keywords:** Al_2_O_3_, ALD, ellipsometry, PE-ALD, XPS

## Abstract

We report on results on the preparation of thin (<100 nm) aluminum oxide (Al_2_O_3_) films on silicon substrates using thermal atomic layer deposition (T-ALD) and plasma enhanced atomic layer deposition (PE-ALD) in the SENTECH SI ALD LL system. The T-ALD Al_2_O_3_ layers were deposited at 200 °C, for the PE-ALD films we varied the substrate temperature range between room temperature (rt) and 200 °C. We show data from spectroscopic ellipsometry (thickness, refractive index, growth rate) over 4” wafers and correlate them to X-ray photoelectron spectroscopy (XPS) results. The 200 °C T-ALD and PE-ALD processes yield films with similar refractive indices and with oxygen to aluminum elemental ratios very close to the stoichiometric value of 1.5. However, in both also fragments of the precursor are integrated into the film. The PE-ALD films show an increased growth rate and lower carbon contaminations. Reducing the deposition temperature down to rt leads to a higher content of carbon and CH-species. We also find a decrease of the refractive index and of the oxygen to aluminum elemental ratio as well as an increase of the growth rate whereas the homogeneity of the film growth is not influenced significantly. Initial state energy shifts in all PE-ALD samples are observed which we attribute to a net negative charge within the films.

## Introduction

Thin aluminum oxide (Al_2_O_3_) layers deposited by atomic layer deposition (ALD) have been investigated for several applications like surface passivation or encapsulation in organic and inorganic photovoltaic devices [1–2], interfacial buffering for high-k dielectrics [3–4], organic memories [5], and nano-laminates [6] as well as work function modification [7], gas diffusion barrier [8] or corrosion protection [9]. Recently, there is a growing activity in covering photo-electrodes or electrodes by ultra-thin Al_2_O_3_ ALD layers for electrochemical energy generation and storage systems [10] in order to enhance the efficiency and durability of such devices. This includes for example solar energy conversion systems like dye sensitized solar cells [11–12] and water splitting devices [13] or lithium ion batteries [14]. Here, in particular the excellent conformability of ALD growth over high surface area materials and its uniformity and self-termination [15] were beneficially applied. Furthermore, Al_2_O_3_ ALD layers have shown their ability as gate dielectrics for future graphene based electronics [16].

The most commonly used ALD sequence for thermal ALD (T-ALD) is the pulsed alternation of trimethyl-aluminum (TMA) as metal source and water as oxygen source, respectively [1,15,17]. Within the last decade the research have been extended to plasma enhanced ALD (PE-ALD) in which the H_2_O as oxygen source is replaced by a plasma exposure (O_2_, O_3_) [1,17–18]. Caused by the higher reactivity of the plasma generated oxygen radicals the PE-ALD extends the capabilities of ALD such as improved film quality, increased flexibility in process conditions [17–18], and is in particular preferred over thermal ALD for lower substrate temperatures due to lower impurity levels [1,18]. The latter allows further the ALD use in organic and in particular flexible electronic applications or on thermally fragile substrates [2,8,15,18].

Recently the Kessels group has reviewed the state of the art of plasma-assisted ALD [18] and surface passivation schemes of Al_2_O_3_ prepared by ALD [1]. This group has reported also on the modeling of reaction regimes influencing the conformality of the PE-ALD process [19]. Herein the typical parameters like growth rate per cycle (GPC), density and refractive index were determined by ellipsometry whereas the elemental composition was mostly deduced from Rutherford Backscattering Spectrometry (RBS). The influence of the substrate temperature onto these parameters was discussed, also when a commercial 200 mm ALD reactor was used [20]. However characterizations based X-ray photoelectron spectroscopy (XPS) in dependence of the substrate temperature are not shown in that reviews. Furthermore to our knowledge there seems to be a lack in reports about dielectric parameters in dependence of the substrate temperature for PE-ALD as mostly comparisons are given at fixed temperatures [1,21] or only for T-ALD samples [22].

In this paper we show a comparison of Al_2_O_3_ samples prepared by T-ALD and PE-ALD respectively based on ellipsometry and on X-ray photoelectron spectroscopy measurements (XPS). The substrate temperature in the PE-ALD process was varied from 200 °C downwards to room temperature (rt). The Sentech ALD reactor system is applicable for both processes (see Experimental section). In that way we are able to investigate samples which are produced in the same chamber avoiding the influence which might be caused by variations of different ALD systems. In the first part we evaluate the newly developed SENTECH’s SI ALD LL system by comparison of homogeneity, GPC, and refractive index with recently reported values in the literature whereas in the second part the oxygen to aluminum (O/Al) ratio and carbon contaminations are discussed. Dielectric parameters of these films will be discussed elsewhere [23].

## Results and Discussion

### Thickness and homogeneity (ellipsometry)

**Figure 1:**
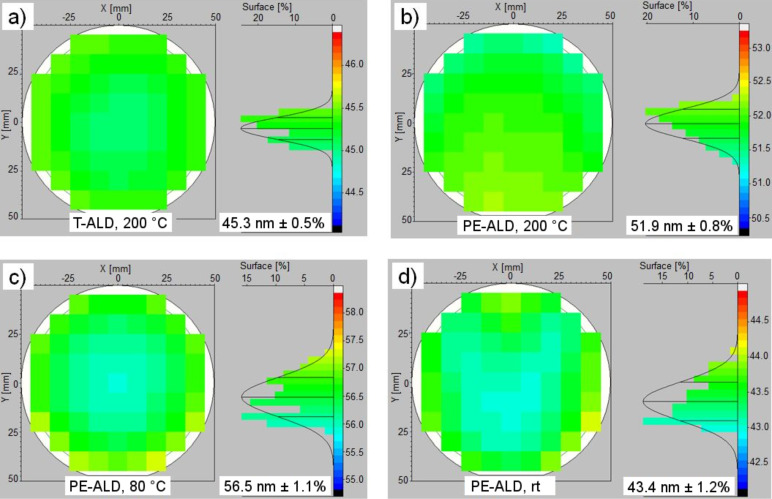
Thickness distributions of T-ALD at 200 °C (a) and PE-ALD films at 200 °C (b), 80 °C (c) and rt (d). The data were recorded by ellipsometry. The left part of every fraction shows the thickness distribution over 4 inch wafers (Ø = 100 mm) and the right part its statistics. The thickness average and the uniformity of the layer over the 4 inch wafer are given within the graphs.

First, we report on the thickness homogeneity of the T-ALD and PE-ALD layers. Film thickness and refractive index of the deposited layers were determined using a SENTECH SE 800 spectroscopic ellipsometry instrument (for details see experimental section). Figure 1 depicts the thickness distributions of the PE-ALD layers prepared at 200 °C, 80 °C and rt; for comparison a film produced at typical T-ALD conditions at 200 °C is shown.

**Figure 2:**
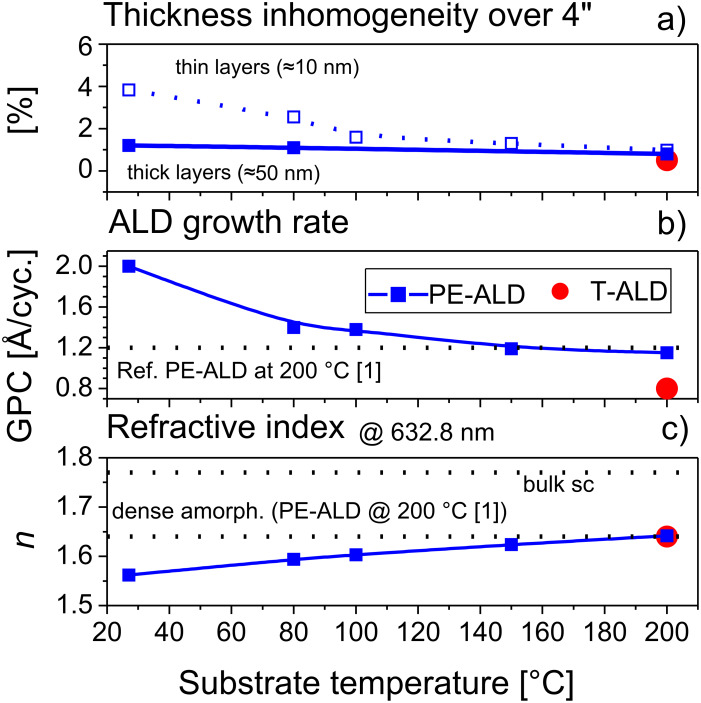
Ellipsometry results showing thickness homogeneity (a), growth rate (b) and refractive index at 632.8 nm (c) in dependence of the substrate temperature of PE-ALD layers (blue squares, filled: ≈50 nm thick films, open: ≈10 nm thick films). The data of the T-ALD film at 200 °C are included (red filled circle). For orientation the reference values of the growth rate and refractive index reported for dense amorphous layers (PE-ALD, 200 °C) in literature [1] and the bulk single crystalline (sc) value of the refractive index are illustrated by dashed lines.

Figure 2a summarizes these homogeneity results in dependence of the substrate temperature. Both, the T-ALD and PE-ALD layers prepared at 200 °C show very good homogeneities with non-uniformities of only ±0.5% and ±0.8%, respectively. For the PE-ALD layers produced at 80 °C (±1.1%) and rt (±1.2%) the values are only slightly increased. For thinner PE-ALD layers (≈10 nm, also shown in Figure 2a) the homogeneity remains approximately the same for *T* > 100 °C. Below this temperature the inhomogeneity increases to ±2.5% and ±3.8% at 80 °C and rt, respectively. Assuming the same roughness in the thicker and thinner layers at the same process temperature, the influence of the roughness on the thickness distribution is increased for the thinner layer. Therefore we argue that at lower temperatures the roughness is increased compared to the layers at *T* > 100°C.

Parasitic chemical vapour deposition (CVD) like reactions due to remaining TMA precursor within the reactor caused by not optimal purge times as well as a radial non-uniformity of the plasma species are believed to be responsible for the thickness non-uniformity in the PE-ALD process [1].

### Growth rate and refractive index (ellipsometry)

The growth rate per cycle and the refractive index at 632.8 nm wavelength are deduced from the ellipsometry data and plotted versus growth temperatures in Figure 2b and 2c. For the PE-ALD process the growth rate of the Al_2_O_3_ film increases from 1.2 Å/cycle to 2 Å/cycle, whereas the refractive index decreases from 1.64 to 1.56 when the substrate temperature is reduced from 200 °C down to rt. The data of the PE-ALD at 200 °C and also the temperature dependency of the growth rate are in very good agreement with values reported by the Kessels group [1]. Also the trend of the refractive index is very similar to reported values by the George group [8,15] for the thermal ALD in the temperature range between 33 °C and 177 °C. For comparison, our thermal ALD procedure for Al_2_O_3_ (200 °C) delivers a growth rate of 0.8 Å/cycle and a refractive index of 1.64, these value are also included in Figure 2.

The observed reduction of the refractive index at lower temperatures corresponds to a slightly reduced mass density [8,15,18]. This might be also partly responsible for the increased GPC values. However, the main driving force for the higher GPC at lower temperatures is attributed to an increased incorporation of aluminum atoms into the layers due to a higher surface density of hydroxyl groups as the dominant adsorption sites for TMA [18]. At higher temperatures thermally activated dehydroxylation reactions occur and the GPC decreases [1]. In addition the CVD parasitic reactions mentioned above may lead to the dissociation of the TMA precursor resulting in higher GPC values [1].

### Oxygen to aluminum elemental ratios (XPS)

**Figure 3:**
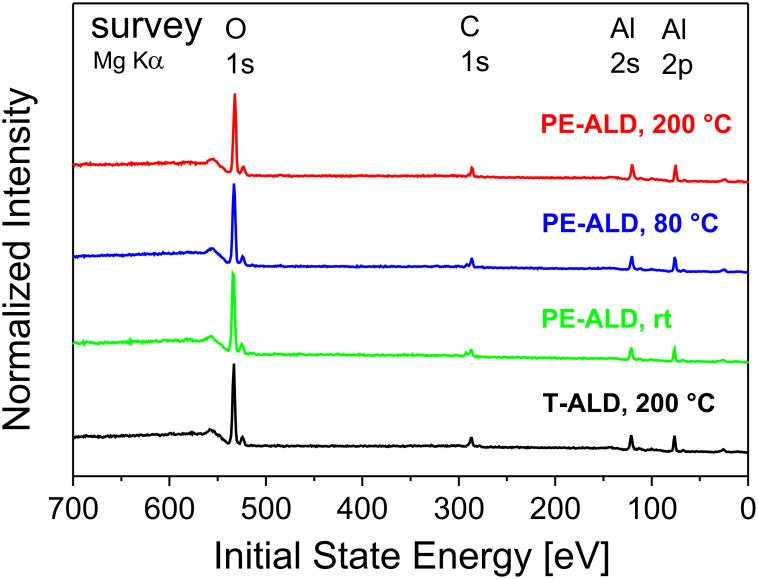
XPS survey spectra (Mg Kα) of the PE-ALD layers deposited at 200 °C (red curve), 80 °C (blue) and rt (green) and the T-ALD at 200 °C (black). The main core levels are labeled. For better comparison the data are normalized to each O1s peak maximum and separated vertically, but shown in the same scaling.

The chemical composition of the Al_2_O_3_ films prepared at different temperatures was investigated by XPS. Based on the general trends reported above, here the PE-ALD layers at 200 °C, 80 °C and rt as well as the T-ALD sample (200 °C) were measured. The XPS survey spectra (Figure 3) of PE-ALD samples (200 °C, 80 °C, rt) and of the T-ALD (200 °C) sample depict mainly Al and O contributions but also carbon contamination. The latter will be discussed below.

Now we focus on the details of the XPS analysis, in particular on the values of the observed peak positions, the carbon content, the contributions in the peak profiles of the Al2p and O1s core levels. We also discuss the origin of the observed peak shifts.

#### Peak positions

**Figure 4:**
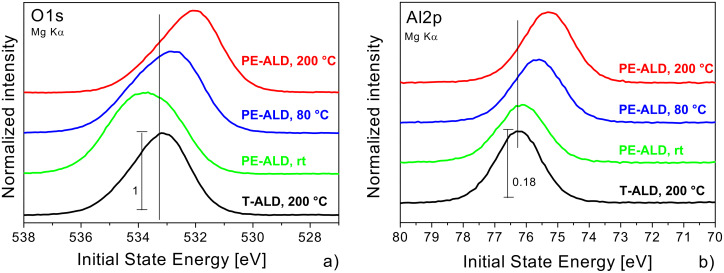
O1s (a) and Al2p (b) core level spectra (Mg Kα) of the PE-ALD layers deposited at 200 °C (red curve), 80 °C (blue) and rt (green) and the T-ALD at 200 °C (black). The data are normalized to each O1s peak maximum and separated vertically, but shown in the same scaling. The IS energy is referred to the Fermi energy.

In Figure 4a and 4b we show the detailed spectra of the O1s and Al2p core levels. First we notice that all observed peak positions appear at very high initial state (IS) energy values. Second, the observed values depend significantly on the preparation conditions and vary within 1 to 1.5 eV. The peak maxima of the O1s and Al2p core levels of the T-ALD sample appear at 533.2 eV and 76.3 eV IS energy. It is obvious that the positions of the peak maxima are shifted towards lower IS energy in both, the O1s and the Al2p data for the PE-ALD samples, except for the O1s of the rt sample discussed below. The observed energy values for the individual core levels and the corresponding FWHM are listed also in Table 1 for the individual samples.

**Table 1:** Summary of the data determined in this contribution for PE-ALD layers at (200 °C, 80 °C, rt) and the T-ALD film at 200 °C. The XPS data shown here are based on Mg Kα excitation.

ALD – Process		T	PE	PE	PE
Temperature [°C]		200	27 (rt)	80	200

Thickness Inhomogeneity [%]	≈50 nm	0.5	1.2	1.1	0.8
	≈10 nm	n.d.	3.8	2.5	0.98
Growth rate [Å/cycle]		0.8	2.0	1.4	1.2
Refractive index		1.64	1.56	1.59	1.64
O/Al ratio (XPS)		1.46	1.20	1.32	1.47
C/Al (EDX)		n.d.	0.22	0.16	0.07
C (XPS) [%]		11	14	14	8
Al2p peak position/FWHM [eV]		76.3/1.7	76.1/1.8	75.6/1.9	75.3/1.9
O1s peak position/FWHM [eV]		533.2/2.4	533.7/3.0	532.8/2.9	532.1/2.6
C1s peak position/FWHM [eV]		286.9/1.8	287.1/1.9	286.5/1.9	286.2/2.0

#### Carbon contributions

**Figure 5:**
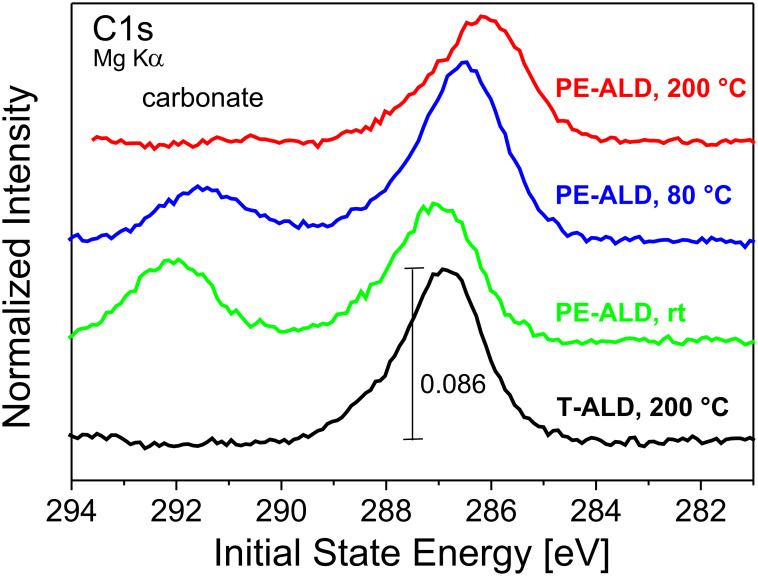
C1s core level spectra (Mg Kα) of the PE-ALD layers deposited at 200 °C (red curve), 80 °C (blue) and rt (green) and the T-ALD at 200 °C (black). The data are normalized to each O1s peak maximum and separated vertically, but shown in the same scaling. The energy is referred to the Fermi energy.

It should be noted that the generally used approach to refer the IS energy to the position of the C1s contribution cannot be applied here. One reason is that the carbon species are not associated with adsorbed methyl groups (284.5 eV) or adsorbed hydro-carbons (285 eV) but are inserted in an oxidic matrix. The formation of C–O bonds results in core level energies at around 286 eV (Figure 5). Second, the peak position of the C1s (Figure 5) varies in the same way as that of the Al2p and the O1s core levels. This indicates that all peak positions are shifted due to the individual preparation conditions.

**Figure 6:**
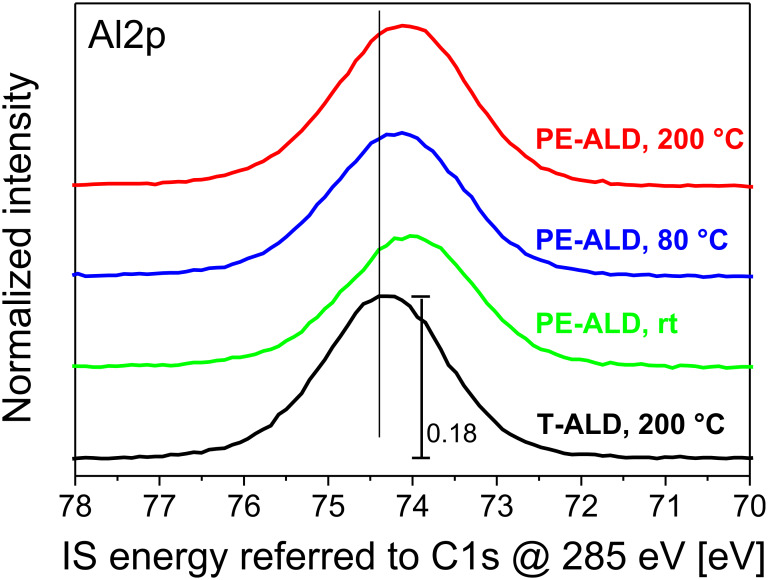
Al2p core level spectra (Mg Kα) of the PE-ALD layers deposited at 200 °C (red curve), 80 °C (blue) and rt (green) and the T-ALD at 200 °C (black) when referred to a fixed C1s peak position of 285 eV for every sample. The data are normalized to each O1s peak maximum and separated vertically, but shown in the same scaling.

In case that the peak positions would be referred to C1s positions at a fixed energy (e.g., 285 eV) we would still observe a shift in the IS energies of both the O1s and Al2p core levels. This is exemplarily illustrated in Figure 6 for the Al2p. All PE-ALD samples exhibit a similar remaining shift of about 400 meV. This we attribute to fixed oxide charges (see below).

Also, due to the fact that we observe significant C1s intensities – we have to consider the contributions from carbonate species [24]. These are incorporated by the precursor side groups which do not desorb completely during the purging periods. The combustion like character of the PE-ALD process yields also COO side products [1,18].

#### Peak profiles

The profiles and FWHM of the core levels can be analyzed to give information about the chemical neighborhood of the individual elements. The data in Figure 4 indicate that both, the Al2p and the O1s levels are rather broad and have some asymmetric profile. Only for the T-ALD sample the shape of the Al2p is rather symmetric and can be decomposed by one single peak which we assign to Al–O [1,24–25]. In contrast, the O1s signal exhibits a shoulder towards higher IS energies. For the PE-ALD samples the line width is broader, in general, and the asymmetries are more pronounced.

We attribute the broadening to the existence of hydroxyl (OH) groups [24] which are incorporated within the films by the usage of the H_2_O oxidant in T-ALD or the higher oxidation potential of the PE-ALD process causing other H_2_O side products [1,18].

**Figure 7:**
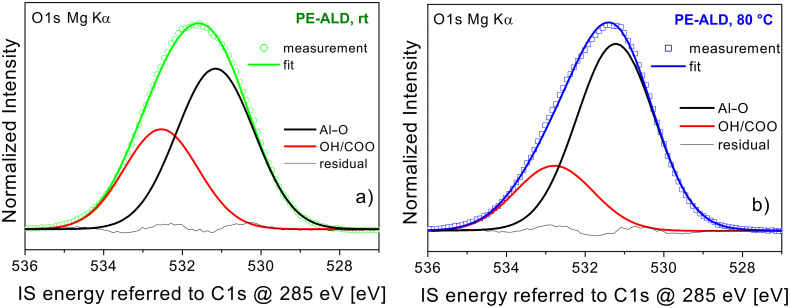
O1s core level spectra (Mg Kα) of the PE-ALD samples prepared at rt (a) and 80 °C (b) substrate temperature. The peak decomposition into Al–O (black curves) and OH/COO components (red) are indicated. The measured data are given by green hollow circles (rt) and blue hollow squares (80 °C) whereas the resulting fitting curve is shown in corresponding line colors (residual: thin black line). The data are normalized to the measured O1s peak maximum of each sample and both diagrams are shown in an identical scale. The IS energy is referred to fixed C1s levels for every sample at 285 eV.

In Figure 7 we have analyzed the profiles of the O1s signals of the PE-ALD samples at rt (Figure 7a) and at 80 °C (Figure 7b) in more detail. In order to allow a comparison to literature values the spectra are referred in this case to fixed C1s core levels. Here the main signal arising from the Al–O bonds [24–25] is found at 531.2 eV for both samples. The second contribution caused by the additional hydroxyl and carbonate species [24] appear at 532.6 eV and 532.7 eV, respectively.

Our assignment of the peak positions is for thick Al_2_O_3_ films where the screening of the photo-excited hole is by the electrostatic potentials of the oxide neighbors while screening from the substrate is negligible [26]. It should be mentioned that these values may differ from those of ultra-thin Al-oxide films reported elsewhere [1,24]. We should emphasize again that the individual IS energy positions for the main Al–O signal depends on the preparation condition of the individual films as these influence the dielectric screening significantly [3,6,27].

From the combination of these data with the C1s core level data (Figure 5) it becomes evident that the rt PE-ALD sample has the highest carbonate content leading to distinct contribution at higher IS energy within the O1s core level data. They compete with the above mentioned and below discussed peak shifting to lower IS energy. Therefore the O1s peak maximum of the rt PE-ALD sample is shifted to higher IS energy with respect to the T-ALD 200 °C sample whereas the peak maximum is moved to lower IS energy in the Al2p core level, where the carbonate has no influence. The same fact is due for the 80 °C PE-ALD sample where the O1s peak maxima is almost at the same position like in the T-ALD 200 °C sample but the Al2p exhibits a shift into the same direction like in the other PE-ALD samples.

#### Relative O/Al ratios

**Figure 8:**
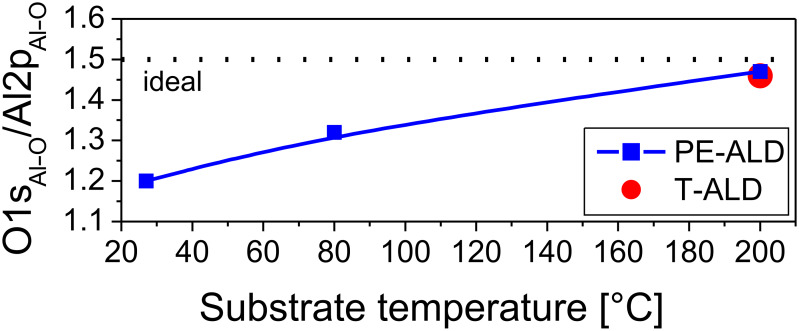
O1s to Al2p elemental ratio versus substrate temperature of PE-ALD layers (blue squares); the data point of the T-ALD film at 200 °C is included (red filled circle). Here, only the Al–O contributions were considered for the analysis. For orientation the ideal stoichiometric value is illustrated by the dashed line.

Based on this data analysis the elemental ratio of oxygen to aluminum was determined. We used the peak areas of the Al–O contributions within the O1s and Al2p core levels (i.e., the contributions assigned to COO and OH groups were not considered). We used the element specific cross sections of 0.063467 and of 0.012295 for O1s and Al2p, respectively [28]. The resulting O1s_Al–O_/Al2p_Al–O_ ratios are plotted versus the substrate temperature in Figure 8. For the samples prepared at 200 °C substrate temperature we find ratios of 1.46 and 1.47 for both, the T-ALD and the PE-ALD samples. These values are close to the stoichiometric value of 1.5. When the temperature in the PE-ALD process is lowered we observe a significant reduction of the O/Al ratio as shown in Figure 8. It points out that at these lower temperatures (80 °C, rt) the oxygen radicals are less efficient in oxidizing the aluminum precursor. This is true also for the chemisorbed organic precursor molecules.

At first sight our results might contradict the composition results of the Kessels group based on RBS data where oxygen rich layers were found at lower temperatures [1,20]. However, we have analyzed here only the Al–O specific contributions. In case that also OH and COO contributions would be considered for the analysis O/Al ratios of 1.9 and 1.8 would be deduced for the PE-ALD samples at rt and 80 °C substrate temperature, respectively, very similar to the results in [20].

#### Influence of fixed charges

Regarding the shifts of the IS energy of the O1s and distinctly in the Al2p core levels of the PE-ALD samples we argue that they might be caused by a net negative fixed charge which is built-up in particular in PE-ALD samples as reported in literature [1,18,22,29]. Our capacitance–voltage measurements on these layers (to be reported elsewhere, [23]) indeed yield a negative fixed charge which is in the range of 0.5 to 5 × 10^12^ cm^−2^ for the PE-ALD samples. For the 200 °C T-ALD sample it is only about 2 × 10^11^ cm^−2^.

Tetrahedral coordinated Al has a charge of −3 [29] and can be counted as an aluminum vacancy which can react with an oxygen atom originating from the SiO_2_ interface resulting in a net negative charge [1,29]. Moreover, oxygen interstitials may be responsible for the net negative fixed charge [1]. However, the microscopic origin of this charge is still under discussion [1]. Therefore, we plan to conduct X-ray absorption as well as resonant photoemission measurements using synchrotron radiation (SR) in near future.

**Figure 9:**
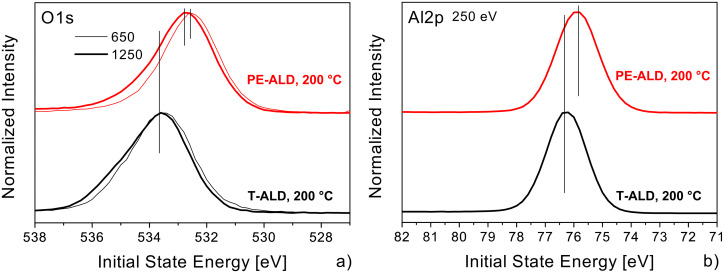
O1s (a) and Al2p (b) core level spectra of the PE-ALD (red curves) and T-ALD layers (black) deposited at 200 °C. These data were recorded with synchrotron excitation. The excitation energies were 650 eV (thick lines) and 1250 eV (thin lines) for the O1s and 250 eV for the Al2p, respectively. The data are normalized to each individual peak maximum and separated vertically, but shown in the same scaling. The IS energy is referred to Fermi energy.

Now we report on a direct comparison of the 200 °C PE-ALD and T-ALD samples. The results are depicted in Figure 9. First, we focused on the line positions of the O1s (Figure 9a) and Al2p (Figure 9b) core levels using SR and observed the same or only slightly shifted IS energies compared to the lab experiments with Mg Kα excitation (compare to Figure 4a and Figure 4b). The IS energy shifts between the PE-ALD and T-ALD samples in the synchrotron experiment are a bit smaller compared to the Mg Kα lab measurement. This might be due to the higher light intensity at the synchrotron which might lead to a filling/defilling of fixed charges. To check further whether the mentioned shifts of the core levels of the PE-ALD samples in comparison to the T-ALD sample originate from some surface bend bending we measured the O1s core level at different excitation energies (Figure 9a). In the T-ALD sample we observe no difference in the line position between the more surface (650 eV) and more bulk sensitive (1250 eV) mode, whereas in the PE-ALD a small shift of 150 meV is within experimental error bars. Therefore we conclude that the shifts in the core levels of the PE-ALD samples are real and not caused by effects like surface bend bending or other experimental uncertainties.

The influence of the substrate is important in these considerations. Bayer et al. report on Al_2_O_3_ films prepared by T-ALD on ITO and found Al2p IS energies between 74.5 eV and 75.5 eV [30]. On ruthenium and ruthenium oxide we found also values between 74.5 eV and 75.0 eV depending on substrate and film thickness [7]. For these conductive oxide substrates the variation in the core level energies may be attributed to surface diploes caused by adsorbed OH groups. This interpretation is based on the fact that there is a change in the energies of about 0.5 eV when spectra are taken after the precursor pulse and after the oxygen pulse.

In contrast, on non-conductive substrates an interface charge is built up. This is proposed based on electrical studies [29,31] which indicated that a fixed charge is generated at the interface of Al_2_O_3_/interfacial SiO_2_. This interface charge induces Coulomb scattering to the surface channel of a field effect transistor which reduces the electron mobility. In our experiments the interface charges cause the additional shift of the core level energies. The amount of such charges varies depending on the individual preparation conditions.

We like to mention that based on our accurate determination of the IS energy we are able to follow shifts in the samples very accurately. In all samples, the IS energy values of the Al2p, O1s, and the C1s level appear to be different. In fact, the variation of the Al2p IS energy values for the highest charge level amounts 75.3 eV and for the lowest charge level we find a value of 76.3 eV. In total, the shifts of about 1 to 1.5 eV are explained by different charge accumulated. In the PE-ALD samples series (Figure 4, Figure 5 and Figure 6) we find an additional trend as upon increasing temperature there is an additional shift of the O1s and Al2p levels with respect to that of the C1s level. This shift is attributed to a structural change in the Al_2_O_3_.

To summarize our XPS data analysis, we have identified fragments of the precursor and the H_2_O oxidant within the films. OH and COO groups appear in the O1s core level, TMA fragments and carbon–oxygen reaction products up to COO show up in the C1s core level. The relative intensity of both is higher in the PE-ALD films because of the higher reactivity of the plasma enhanced mode. The peak positions of all films are influenced by charged species within the films.

### Carbon contamination (EDX, XPS, ellipsometry)

In order to discuss the integration of carbon atoms into the films we conducted energy dispersive X-ray spectroscopy (EDX), XPS C1s core level spectroscopy, and spectroscopic ellipsometry.

**Figure 10:**
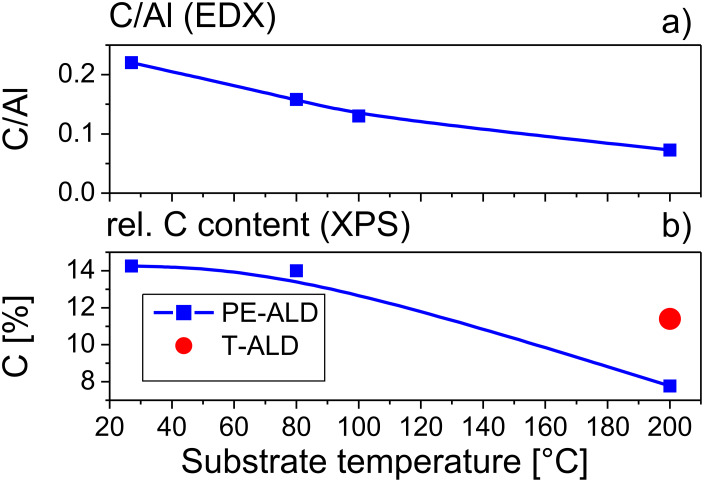
Carbon content within PE-ALD layers (blue squares) versus substrate temperature determined by EDX (a) and XPS (b). For comparison the XPS result of the T-ALD film at 200 °C is included (red filled circle).

The chemical composition of the Al_2_O_3_ films at different temperatures was investigated by EDX. Hereby, the relation between C-atoms and Al-atoms within the films was determined. Figure 10a displays the ratio of carbon to aluminum depending on the substrate temperature of the film. With decreasing deposition temperature, the carbon proportion within the Al_2_O_3_ films increases significantly.

The same trend is observed in the XPS data (refer to Figure 5). Here the C1s peak areas with a cross section of 0.021314 [28] were related to the total sum of the cross section weighted O1s, Al2p and C1s peak areas of every sample. The resulting total carbon concentrations are shown in Figure 10b; the corresponding C1s core level data are presented in Figure 5. Remarkably, the PE-ALD at 200 °C exhibits a clear reduction to around 8% compared to 11% of the T-ALD at this temperature. The lower carbon content of the PE-ALD sample at 200 °C is also evident by a qualitative comparison of the normalized C1s core levels as depicted in Figure 5. At lower temperatures of 80 °C and rt the carbon concentration increases to about 14%. As the carbon concentration shows the same trend as the O/Al ratios reported above we argue that the metal precursor interaction with the substrate is not completed leading to higher carbon and lower oxygen contents.

This fact is further supported by carbonate contributions rising in the C1s core level data of the PE-ALD samples prepared at 80 °C and rt (see Figure 5). In the PE-ALD combustion-like reactions occur with the formation of COO and H_2_O [1] which may also support secondary reaction pathways [18] leading for example to carbonates and carbon contamination.

It has to be pointed out that the XPS measurements were performed ex-situ and the results might be strongly influenced by surface contamination due to the ex-situ handling of the samples in particular in the surface sensitive XPS method. Nevertheless, our elemental composition data confirm findings of other authors based on RBS data [1,20]. In addition, the observed trend of carbon components within the films is similar to the EDX measurements shown above and the infrared data reported now.

**Figure 11:**
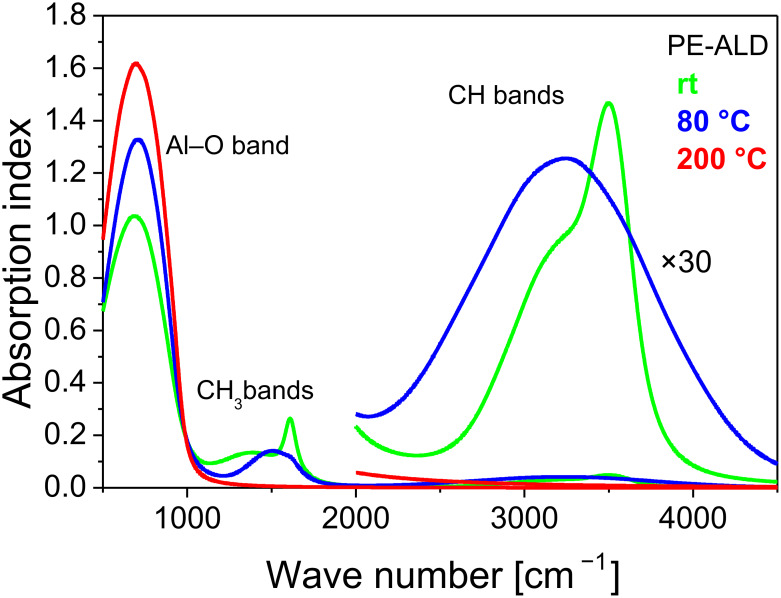
Infrared absorption index data for PE-ALD layers deposited at 200 °C (red curve), 80 °C (blue) and rt (green). The region of the CH bands is magnified (×30).

The incorporation of CH-molecule groups was also monitored by means of infrared spectroscopy. For this purpose, spectroscopic ellipsometry is particularly suitable because hereby the influence of substrate properties can be neglected and no distracting overlays with substrate bands occur and the measured spectra can be directly fitted using an appropriate model. The optical properties of the deposited films were modeled using a Brendel oscillator model. The parameters of the oscillator model are: oscillator frequency, oscillator strength, oscillator damping, and a distribution factor taking into account the influence of surrounding materials of the single oscillator. This model can be applied for all absorbing molecule groups in the Al_2_O_3_ film. In the infrared the thin native oxide film cannot be measured and was neglected. Figure 11 shows the measured spectral dependency between the extinction coefficients (absorption indices) of PE-ALD films with different deposition temperatures. In the graph the positions of oscillation bands caused by Al–O, methyl (CH_3_), and CH groups are indicated. For lower growth temperatures (80 °C, rt) the spectra exhibit distinct contributions of methyl and CH groups indicating not terminated surface reactions of the TMA precursor. At 200 °C no more carbon-based bands are detectable. In reverse, with decreasing concentration of carbon groups, the absorption by the Al–O oscillation band increases indicative for efficient ALD Al_2_O_3_ reaction [15]. These data are in good agreement to the O/Al ratios and the EDX and XPS carbon measurements. However in the 200 °C PE-ALD sample no more CH_x_ bands are detected, whereas the XPS still reflects carbon and carbonate contributions within the films. XPS is a more surface sensitive method than the infrared spectroscopy. Therefore, we conclude that CH_x_ bonds are integrated within the volume of the PE-ALD layers at lower temperatures, whereas carbon contamination and carbonate formation is occurring at the surface.

## Conclusion

Thin homogenous Al_2_O_3_ layers were successfully produced by standard thermal (200 °C) and plasma enhanced ALD in the SENTECH SI ALD LL system at substrates temperatures ranging from 200 °C down to rt. Comparing the 200 °C processes similar refractive indices of 1.64 and oxygen to aluminum elemental ratios near the stoichiometric value of 1.5 were observed. However, the PE-ALD at this temperature exhibits favorably increased growth rates and reduced carbon contaminations. The reduction of the deposition temperature of the PE-ALD down to rt leads to the integration of carbon, COO and CH_x_ compounds. Methyl groups and derivatives of the TMA precursor are integrated into the film due to not completed surface reactions of the aluminum and oxygen precursors. As a result the refractive index and oxygen to aluminum elemental ratio are decreased whereas the growth rate is increased. Nevertheless, the homogeneity of the film growth is not significantly influenced. We conclude that the PE-ALD process at lower temperatures needs therefore an optimization of the cycle and purge time combinations.

Our results contribute to possible deposition on thermally fragile substrates [8,15] and to higher throughput processes in industrial applications [18].

## Experimental

**Figure 12:**
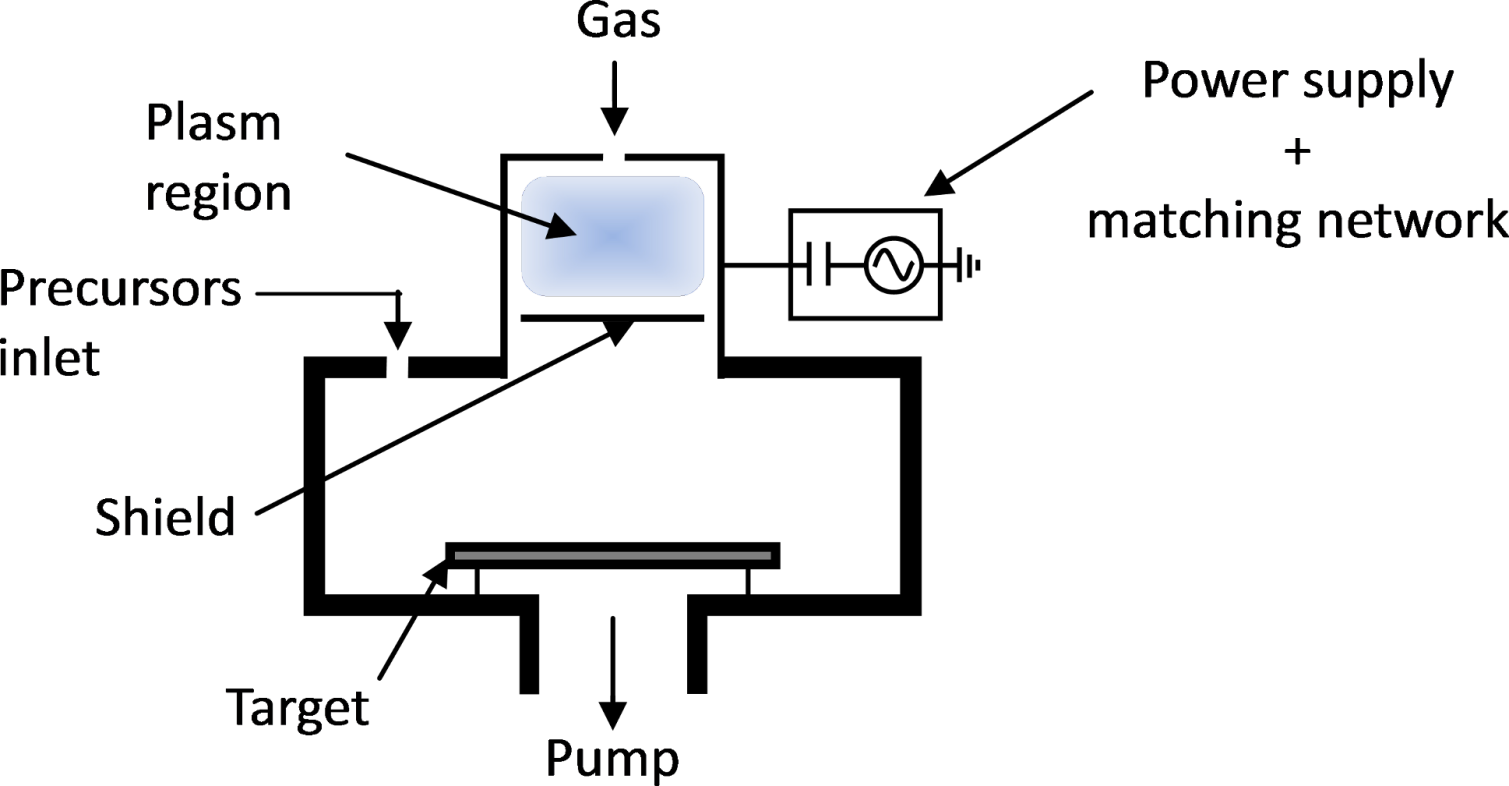
Reactor and CCP source of the SENTECH ALD system: SI ALD LL.

SENTECH’s SI ALD LL system shown schematically in Figure 12 is equipped with a plasma source for PE-ALD processes. The capacitive coupled plasma source (CCP source) was developed by SENTECH Instruments and guarantees a stable pulse operation in ALD cycles. Furthermore, no automatic matching of CCP source during PE-ALD process is needed: the plasma source is pulsed with constant matching parameters during the deposition process. Substrate shuck, reactor and precursor lines are equipped with different heaters. The substrate temperature can be controlled in the range between room temperature and 500 °C, reactor and precursor lines can be heated up to 150 °C and 200 °C respectively. The system consists of 3 precursor lines.

The true remote CCP source, driven by a 13.56 MHz generator, is attached to the upper flange of the reactor. During the deposition process the substrate is placed outside of the plasma generation region; the coated surface is not bombarded with destructive ions and does not see the light from plasma generation region.

PE-ALD Al_2_O_3_ films were deposited on 4” n-type silicon wafers (consisting of a native oxide of approximately 1.5 nm) by the SI ALD LL system at 200 °C, 150 °C, 100 °C, 80 °C, and 27 °C (rt) substrate temperature. Nitrogen (N_2_) with 40 sccm flow was used as carrier gas for TMA. Atomic oxygen was generated by SENTECH’s CCP source. Thereby, a constant oxygen flow rate of 75 sccm was adjusted. The pulse duration of the TMA was 120 ms whereas for the oxygen semi-cycle 5 s (200 °C), 6 s (80 °C) or 7 s (rt) were chosen. The plasma source was run in a pulsed mode as it was operated only during the oxygen step of the ALD cycle with a power of 100 W except for the rt sample (50 W). Process pressure was 20 Pa.

For the T-ALD also N_2_ with 120 sccm flow rate was used as carrier gas for TMA and H_2_O which was applied as oxygen source. Here, the pulse duration was 60 ms for both the TMA and H_2_O. Process pressure was 12 Pa.

Layers of about 50 nm were produced for the ellipsometry investigation of the film properties. To determine film thickness and refractive index spectroscopic ellipsometry (SE 800, SENTECH Instruments GmbH) was used within the UV–vis spectral range. The SENTECH SE 800 is equipped with a scan analyzer for highly accurate spectra. The measurements were performed at an angle of incidence of 70° using a spectral range of 280–850 nm (1.5–4.4 eV). The accumulated spectra were modeled using SpectraRay 3 software. The model layers comprised a Si substrate, a fixed layer of 1.5 nm native SiO_2_ and the deposited Al_2_O_3_ film. The SiO_2_ and the Al_2_O_3_ layers were defined as Cauchy layers. For the detection of CH_x_ compounds within the deposited films spectroscopic ellipsometry in the middle infrared wavelength range (MIR) the SENDIRA ellipsometer from SENTECH Instruments GmbH was performed. A Step scan analyser for high spectroscopic accuracy was used at an angle of incidence of 70°. Modelling was carried out using SpectraRay 3 software. The model layer comprised Al_2_O_3_ on Si-substrate and the layer was modelled over the wave number range of 600 cm^−1^ to 4500 cm^−1^. Additionally, EDX was applied on these samples (≈50 nm).

For XPS measurements Al_2_O_3_ films with a thickness of about 10 nm were prepared in order to avoid charging of the samples. XPS measurements were performed either by Specs Mg Kα source (in the lab) or by synchrotron radiation (undulator beamline U49/2-PGM2 at BESSY-II in Berlin/Adlershof). The data were recorded using semispherical electron analyzers made by Leybold–Heraeus (lab) or Omicron NanoTechnology GmbH (EA125 at BESSY). Both, beam line monochromator and analyzers are controlled for their accuracy in determining the IS energy by running Au4f (87 eV IS energy) spectra at different excitation energies. All spectra shown in this contribution were Shirley background corrected [32]. The kind of normalization of the XPS spectra is given separately in every related figure caption.
